# Medication Use Patterns and Exposure to High-Risk Medications Among Pregnant Women Seeking Counseling from a Teratology Information Service: A Nine-Year Retrospective Study

**DOI:** 10.3390/jcm15207800

**Published:** 2026-10-09

**Authors:** Gülce Sevdar Çeçen, Bilge Çetinkaya Demir, Taha Harun Özdemir, Sinan Çavun, Mustafa Sertaç Yılmaz

**Affiliations:** 1 Department of Pharmacology, Faculty of Medicine, Bursa Uludag University, 16059 Bursa, Türkiye; tahaharunozdemir@uludag.edu.tr (T.H.Ö.); scavun@uludag.edu.tr (S.Ç.); sertacyilmaz@uludag.edu.tr (M.S.Y.); 2 Department of Perinatology, Faculty of Medicine, Bursa Uludag University, 16059 Bursa, Türkiye; drbilgecd@gmail.com

**Keywords:** pregnancy, medication exposure, polypharmacy, Teratology Information Service

## Abstract

**Background:** Medication use during pregnancy is common; however, exposure to medications with potential fetal risks remains an important public health concern. This study aimed to characterize medication use patterns and assess exposure to potentially high-risk medications among women seeking counseling from a Teratology Information Service. **Methods:** This retrospective descriptive study included all consultations provided by a Teratology Information Service between January 2017 and December 2025. Maternal demographic characteristics, gestational age at consultation, medication exposures, and consultation-related data were retrospectively extracted from service records. Medications were classified according to the World Health Organization Anatomical Therapeutic Chemical (ATC) Classification System and the former U.S. Food and Drug Administration (FDA) pregnancy risk categories (A, B, C, D, and X). Vitamin and mineral supplements were analyzed separately. Polypharmacy was defined as the concurrent use of five or more medications. **Results:** A total of 483 consultations were evaluated, including 469 pregnant women and 14 women seeking preconception counseling. The mean maternal age was 31.1 ± 5.5 years, and the mean gestational age at presentation was 9.7 weeks. Most consultations occurred during the first trimester (81.4%). Pregnant women used a mean of 3.88 ± 2.72 medications, and polypharmacy was identified in 31.3% of cases. According to the ATC classification, nervous system drugs were the most frequently used medication group (22.7%), followed by musculoskeletal system drugs (14.7%), alimentary tract and metabolism drugs (13.0%), and anti-infectives for systemic use (11.6%). Among medications classified according to FDA pregnancy risk categories, category C drugs predominated (50.2%). Overall, 57.3% of pregnant women were exposed to at least one category D or X medication. More than half (54.8%) of category X exposures consisted of hormonal preparations. Exposure to at least one category D/X medication was significantly more frequent among women with polypharmacy than among those without polypharmacy (*p* = 0.006). **Conclusions:** High-risk medication exposure was common among pregnant women seeking teratogenic risk counseling, particularly in those with polypharmacy. These findings highlight the importance of comprehensive medication review and individualized risk assessment during pregnancy and emphasize the value of Teratology Information Services in supporting evidence-based medication counseling.

## 1. Introduction

Medication use during pregnancy is common, with the majority of pregnant women using at least one medication during gestation [1,2]. The increasing prevalence of chronic maternal conditions, together with advancing maternal age, has contributed to a growing need for pharmacological treatment during pregnancy [2,3]. Although appropriate pharmacotherapy is often essential for maintaining maternal health and preventing adverse pregnancy outcomes, increasing medication use may also contribute to polypharmacy, particularly among women with chronic conditions requiring ongoing treatment [4]. Polypharmacy is commonly defined as the concurrent use of five or more medications, including in studies involving pregnant populations [4,5,6]. In a large population-based study involving approximately 1.5 million pregnancies, multiple medication prescribing was more common among women with multimorbidity, highlighting the additional complexity of pharmacotherapy in pregnant women with chronic conditions [4,6]. As the number of medications increases, the risk of inappropriate or high-risk prescribing, drug–drug interactions, and medication-related adverse events may also increase [4,6]. Furthermore, polypharmacy during pregnancy may raise concerns regarding both maternal and fetal safety. A recent prospective observational study involving antenatal and postnatal women reported an independent association between polypharmacy and maternal complications [7].

Despite the widespread use of medications during pregnancy, pregnancy-specific safety data remain limited for many drugs, making individualized benefit–risk assessment an essential component of therapeutic decision-making. Historically, the U.S. Food and Drug Administration (FDA) classified medications into pregnancy risk categories A, B, C, D, and X [8]. These categories were intended to summarize the available evidence regarding fetal risk and the potential benefits of medication use during pregnancy. In particular, categories D and X indicated evidence of fetal risk, although their clinical implications differed: category D medications could be used in certain circumstances when the potential benefits justified the risks, whereas category X medications were considered contraindicated during pregnancy [8]. Although this classification has been replaced by the Pregnancy and Lactation Labeling Rule (PLLR), it provides a framework for describing historical medication exposure patterns.

In this context, Teratology Information Services (TISs) play an important role by providing evidence-based, individualized counseling regarding medication exposures and their potential maternal and fetal risks [9,10].

Data from TISs also provide valuable insight into real-world medication use and the characteristics of women seeking teratogenic risk assessment. However, data integrating medication use patterns, polypharmacy, chronic diseases, and exposure to potentially high-risk medications among women seeking teratogenic risk counseling remain limited.

Therefore, this study aimed to characterize medication utilization patterns, polypharmacy, chronic diseases, and exposure to former FDA pregnancy risk categories among women referred to a Teratology Information Service between 2017 and 2025. Specifically, we evaluated maternal demographic and pregnancy-related characteristics, the prevalence and distribution of chronic diseases, the number and types of medications used, and the frequency of polypharmacy. Medication exposures were further characterized according to the Anatomical Therapeutic Chemical (ATC) classification system and the former FDA pregnancy risk categories, with particular attention to exposure to category D and X medications. We also examined the association between polypharmacy and patient-level exposure to at least one category D or X medication, as well as differences in the distribution of medication-level FDA pregnancy risk categories according to chronic disease status.

## 2. Materials and Methods

### 2.1. Study Design and Populations

This retrospective descriptive study included all women who presented to the Maternal Fetal Medicine Outpatient Clinic of Bursa Uludag University and were referred to the Department of Medical Pharmacology, Bursa Uludag University Faculty of Medicine, for teratogenic risk assessment or medication counseling between January 2017 and December 2025. Both pregnant women and women seeking preconception counseling were eligible for inclusion. No specific exclusion criteria were applied. Each consultation corresponded to a unique woman, and no repeated consultations from the same individual were included in the dataset. Accordingly, the study included 483 unique women, comprising 469 pregnant women and 14 women seeking preconception counseling.

The study was approved by the Health Sciences Ethics Committee of Bursa Uludag University Faculty of Medicine (Date: [26 August 2026]; Number: [2026/477/14-46]). Information regarding maternal age, gestational week, indication for consultation, preconception or pregnancy status, underlying diseases, and medication exposure was retrospectively extracted from the consultation records prepared during routine teratology counseling. Gestational age at the time of consultation was calculated based on the first day of the last menstrual period (LMP) when available; when the LMP was unknown, gestational age determined by ultrasonographic assessment was used.

All medications were classified according to the Anatomical Therapeutic Chemical (ATC) Classification System developed by the World Health Organization [11]. Drugs were categorized according to their anatomical main group, therapeutic subgroup, pharmacological subgroup, chemical subgroup, and chemical substance using the five-level ATC classification system.

In addition, medications were classified according to the former United States Food and Drug Administration (FDA) pregnancy risk classification system (categories A, B, C, D, and X). Briefly, category A indicates that controlled studies in pregnant women have not demonstrated fetal risk; category B indicates no evidence of fetal risk in animal studies, although adequate human studies are lacking, or that animal findings of risk have not been confirmed in human studies; category C indicates that fetal risk cannot be ruled out and potential benefits may justify use; category D indicates positive evidence of human fetal risk, although potential benefits may warrant use in certain circumstances; and category X indicates that fetal risks outweigh any potential benefits and use during pregnancy is contraindicated [8]. Although this classification has been replaced by the PLLR, it was used in the present study to ensure consistent categorization throughout the study period (2017–2025) and because the former FDA pregnancy categories continue to be included in the Summary of Product Characteristics (SmPCs) of many medications marketed in Türkiye. Vitamin and mineral supplements were analyzed separately and were excluded from the FDA pregnancy risk category analysis.

### 2.2. Statistical Analysis

Statistical analyses were performed using jamovi version 2.4.8 (the jamovi project). Continuous variables were summarized as mean ± standard deviation (SD) and median (range), as appropriate, while categorical variables were presented as frequencies and percentages. Associations between categorical variables, including polypharmacy status and exposure to former FDA pregnancy category D/X medications, as well as chronic disease status and FDA pregnancy risk categories, were evaluated using Pearson’s chi-square test. A two-sided *p* value < 0.05 was considered statistically significant. The association between polypharmacy and exposure to at least one category D/X medication was evaluated at the individual-woman level. In contrast, the comparison of FDA pregnancy risk category distributions between women with and without chronic diseases was performed at the medication-exposure level, with each recorded medication exposure treated as a separate observation.

## 3. Results

### 3.1. Study Population and Baseline Characteristics

A total of 483 consultations were evaluated between 2017 and 2025. Fourteen of these patients (2.9%) were planning to become pregnant. The mean age of the study population was 31.1 ± 5.5 years, with a median of 31 years (range: 16–46). Only 0.4% of the patients were younger than 18 years, while 27.7% were aged 35 years or older.

The mean gestational age at presentation was 9.69 ± 3.94 weeks. The majority of patients presented during the first trimester (81.4%), followed by the second trimester (18.6%), while no patients presented at ≥28 weeks of gestation.

The mean number of drugs used was 3.88 ± 2.72, with a median of 3 (range: 1–16). Polypharmacy, defined as the use of five or more drugs, was observed in 31.3% of patients. The baseline characteristics of the study population are summarized in Table 1.

**Figure 1:**
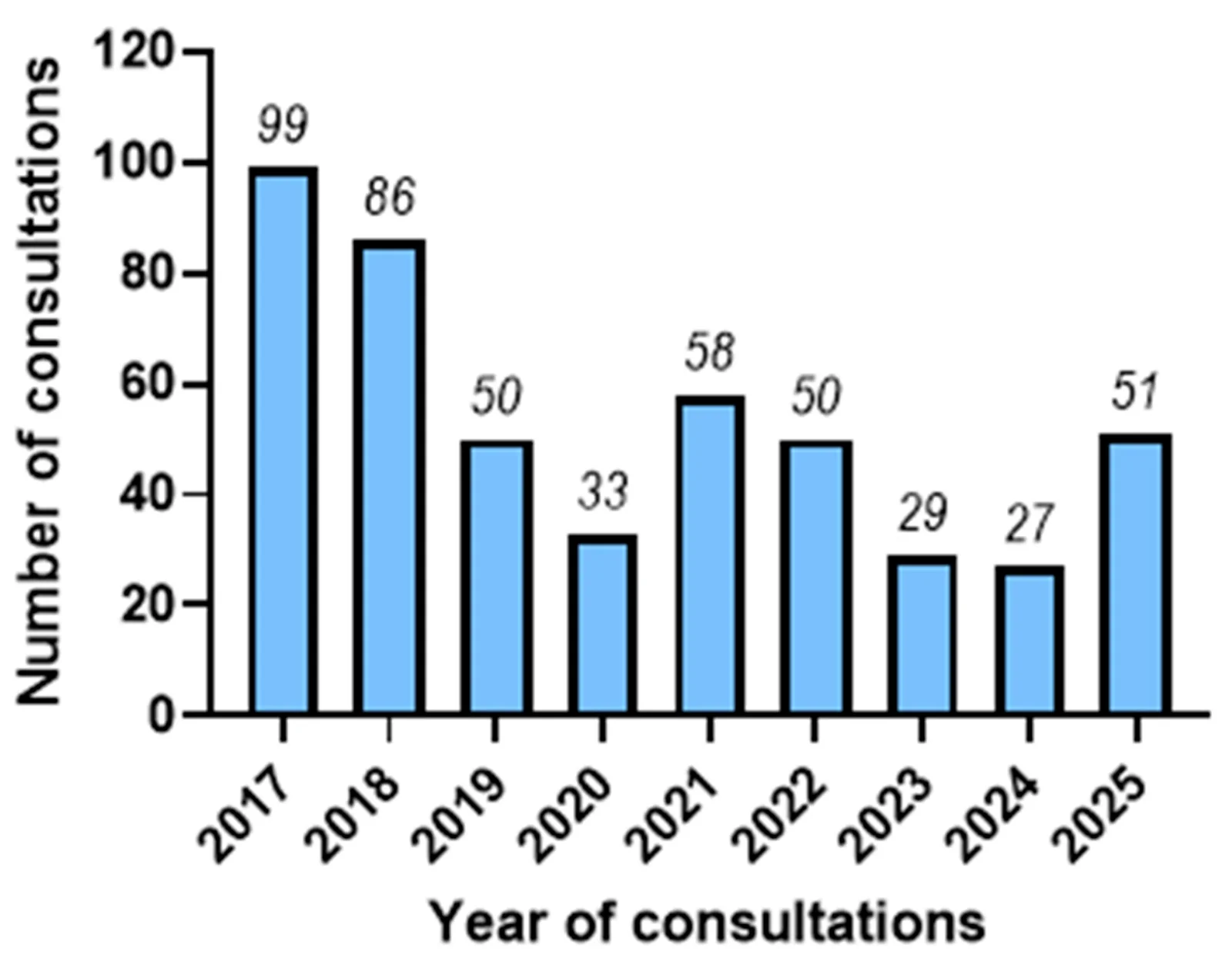
Distribution of consultations by year.

The distribution of patients by year is presented in Figure 1. The highest number of patients was observed in 2017 (*n* = 99, 20.5%) and 2018 (*n* = 86, 17.8%), followed by a decline in subsequent years, with the lowest numbers in 2023 (*n* = 29, 6%) and 2024 (*n* = 27, 5.6%). A slight increase was observed again in 2025 (*n* = 51, 10.6%).

### 3.2. Comorbidities

**Figure 2:**
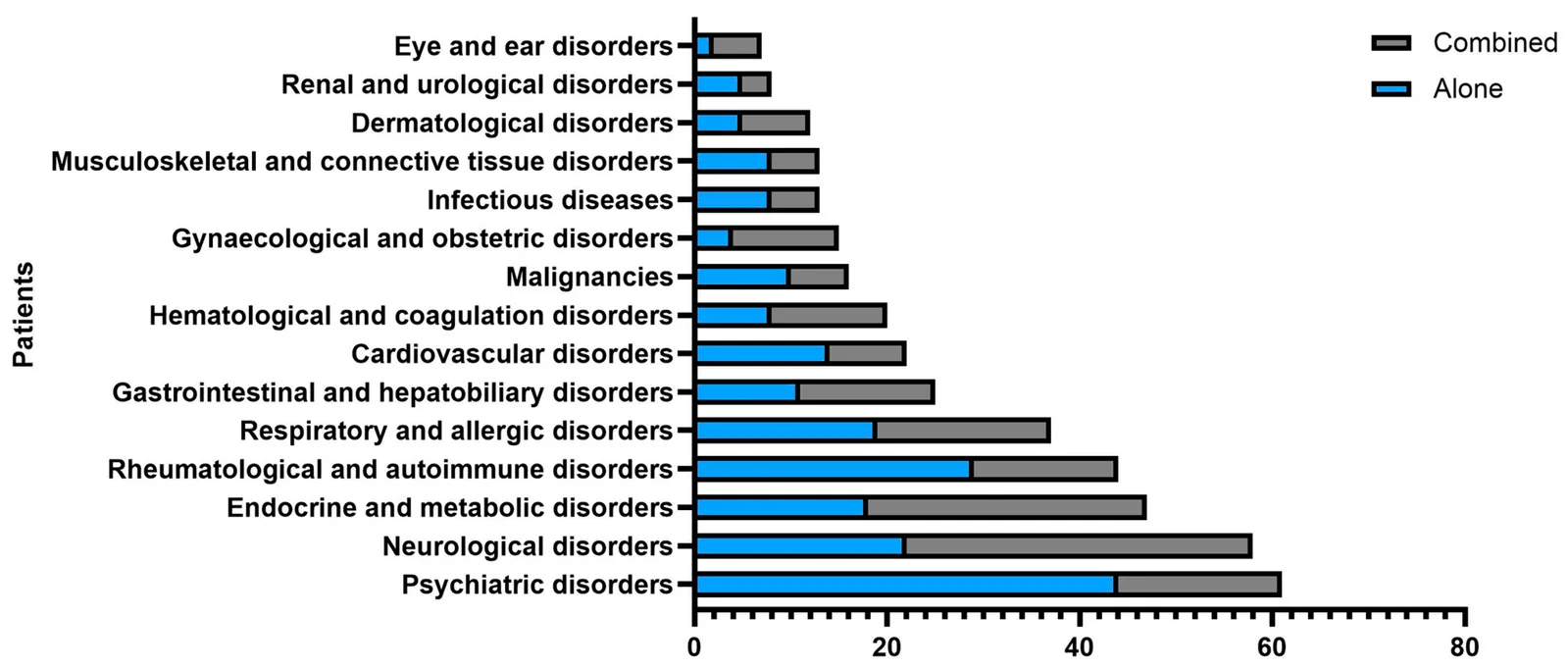
Distribution of chronic disease groups among pregnant women with documented chronic diseases. Bars represent the total number of patients within each disease category. Patients with multiple chronic diseases could contribute to more than one disease group. Blue bars indicate isolated chronic diseases, whereas dark gray bars indicate chronic diseases occurring in combination with at least one additional chronic condition.

Among the 469 pregnant women, 312 (66.5%) had at least one documented chronic disease, whereas 157 (33.5%) had no documented chronic disease. Among patients with chronic diseases, psychiatric disorders (*n* = 61), neurological disorders (*n* = 58), endocrine and metabolic disorders (*n* = 47), and rheumatological and autoimmune disorders (*n* = 44) were the most frequently represented disease groups. The distribution of chronic disease groups is presented in Figure 2.

### 3.3. Distribution of Drugs According to ATC Classification

According to the ATC classification, nervous system medications (group N) were the most frequently used (22.7%), followed by medications targeting the musculoskeletal system (group M; 14.7%), alimentary tract and metabolism (group A; 13.0%), and anti-infectives for systemic use (group J; 11.6%). Within group N, psychoanaleptics (7.4%) and analgesics (5.3%) were the most commonly used subgroups. Within group M, anti-inflammatory and antirheumatic drugs predominated (11.7%). In group A, drugs for acid-related disorders (3.9%) and functional gastrointestinal disorders (2.3%) were the most frequently used, whereas antibacterials for systemic use constituted the largest subgroup within group J (8.8%). Other ATC groups included medications for the respiratory system (7.5%), genitourinary system and sex hormones (7.0%), cardiovascular system (4.7%), and blood and blood-forming organs (4.7%). The distribution of medications according to the ATC classification is presented in Table A1 (Appendix A).

### 3.4. Most Frequently Used Medications

After grouping drugs according to their main active ingredient, the most frequently used drugs were dexketoprofen (*n* = 57, 3.2%), paracetamol (*n* = 56, 3.1%), ibuprofen (*n* = 41, 2.3%), and amoxicillin (*n* = 41, 2.3%), followed by acetylsalicylic acid (*n* = 34, 1.9%), medroxyprogesterone (*n* = 30, 1.7%), diclofenac (*n* = 29, 1.6%), sertraline (*n* = 25, 1.4%), levothyroxine (*n* = 22, 1.2%), and pantoprazole (*n* = 20, 1.1%).

### 3.5. Former FDA Pregnancy Risk Categories

**Figure 3:**
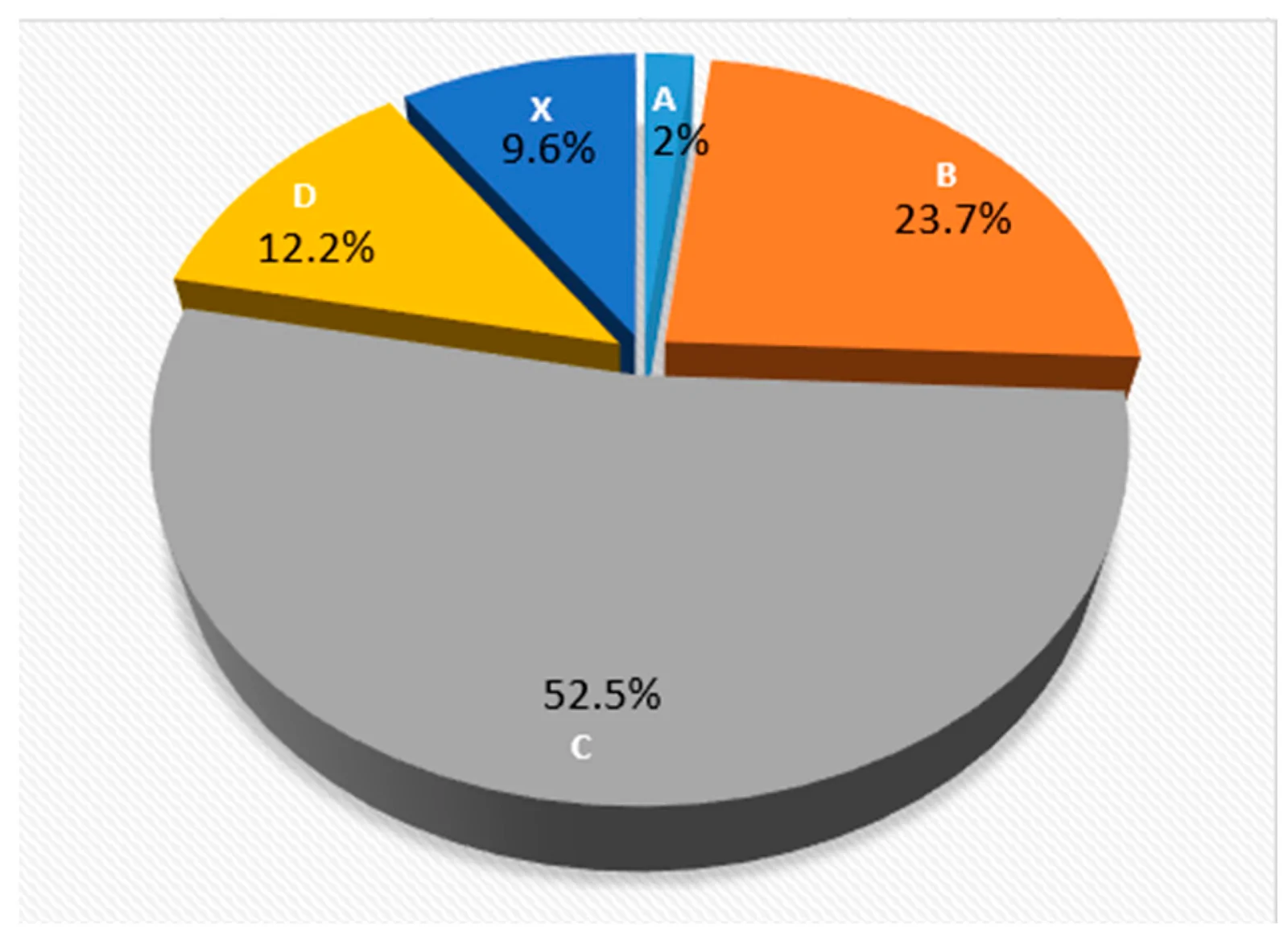
Distribution of medications according to the former FDA pregnancy risk categories. Vitamins and mineral supplements were excluded from the analysis. A: Controlled studies in pregnant women have not demonstrated fetal risk; B: No evidence of fetal risk in humans; C: Fetal risk cannot be ruled out; D: Positive evidence of human fetal risk, although potential benefits may justify use in certain circumstances; X: Contraindicated in pregnancy because fetal risks outweigh any potential benefits.

Vitamins and other products, which accounted for 4.4% of all recorded exposures, were analyzed separately and excluded from the FDA pregnancy risk category analysis. Among the remaining medication exposures, category C accounted for the largest proportion (52.5%), followed by category B (23.7%), category D (12.2%), category X (9.6%), and category A (2.0%) (Figure 3).

Vitamin and supplement products were analyzed separately. The most frequently reported were mineral supplements (*n* = 16, 20.0%), B-complex vitamins (*n* = 15, 18.8%), vitamin D preparations (*n* = 11, 13.8%), and folic acid-containing products (*n* = 10, 12.5%). Other supplements accounted for 8.8% (*n* = 7), while herbal and alternative products and multivitamin preparations each accounted for 7.5% (*n* = 6). COVID-19 vaccines accounted for 5.0% (*n* = 4) and were evaluated separately. Unidentified over-the-counter supplements for which the exact content could not be determined accounted for 3.8% (*n* = 3), and vitamin C preparations accounted for 2.5% (*n* = 2).

### 3.6. Exposure to High-Risk Drugs (Category D and X)

**Figure 4:**
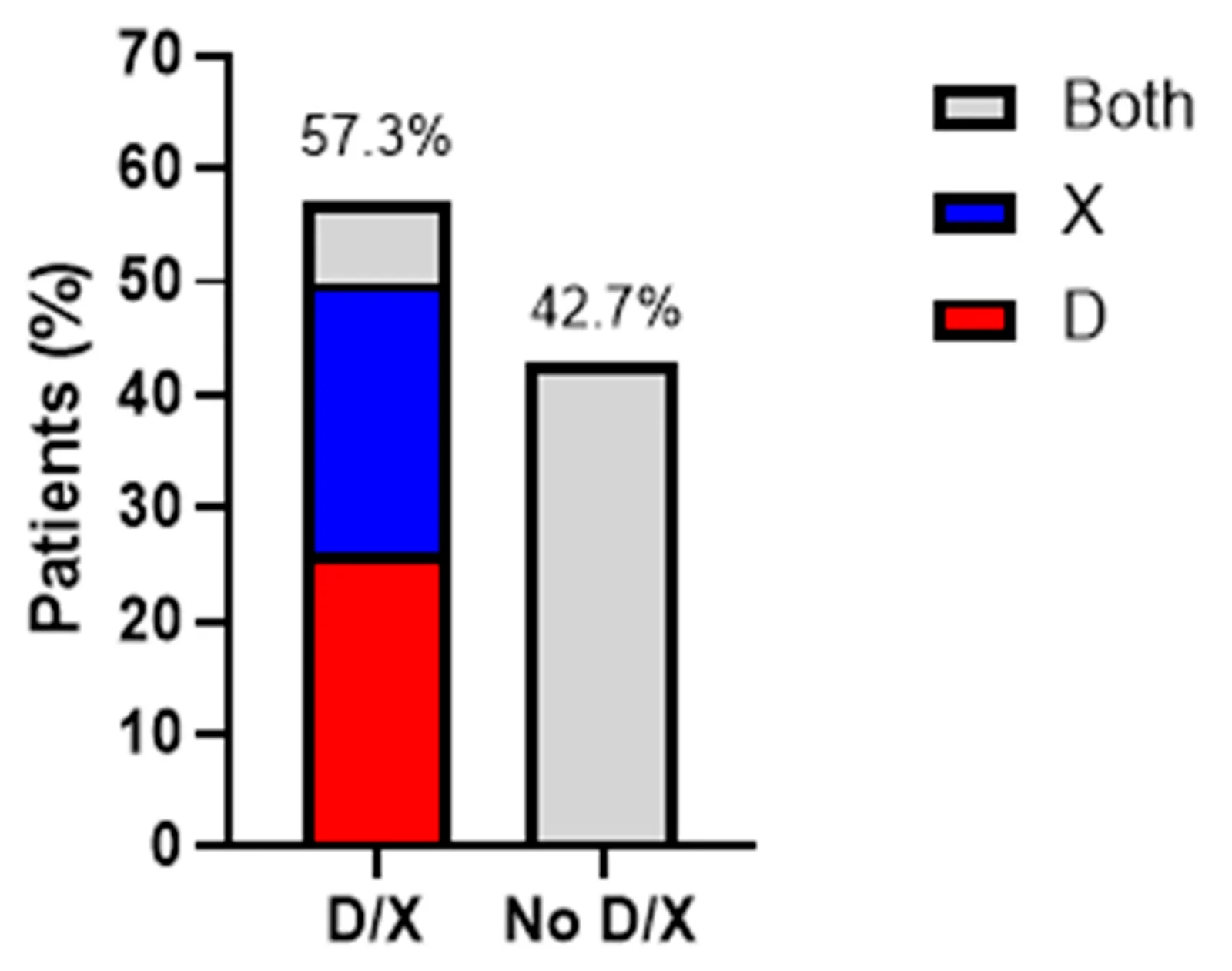
Proportion of pregnant women exposed to at least one medication classified as former FDA pregnancy category D or X. Exposure to categories D and X was not mutually exclusive.

Overall, 57.3% of pregnant women were exposed to at least one FDA category D or X drug, whereas 42.7% had no exposure to these high-risk medications (Figure 4). When evaluated separately, 33.3% of patients were exposed to at least one category D drug and 30.5% to at least one category X drug; these proportions were not mutually exclusive.

The rate of exposure to at least one category D/X drug was similar between the first (56.8%) and second (59.5%) trimesters.

Among category X drugs, the majority (54.8%) were hormonal agents, predominantly the sex hormones group (ATC G03), whereas 45.2% were non-hormonal drugs.

**Figure 5:**
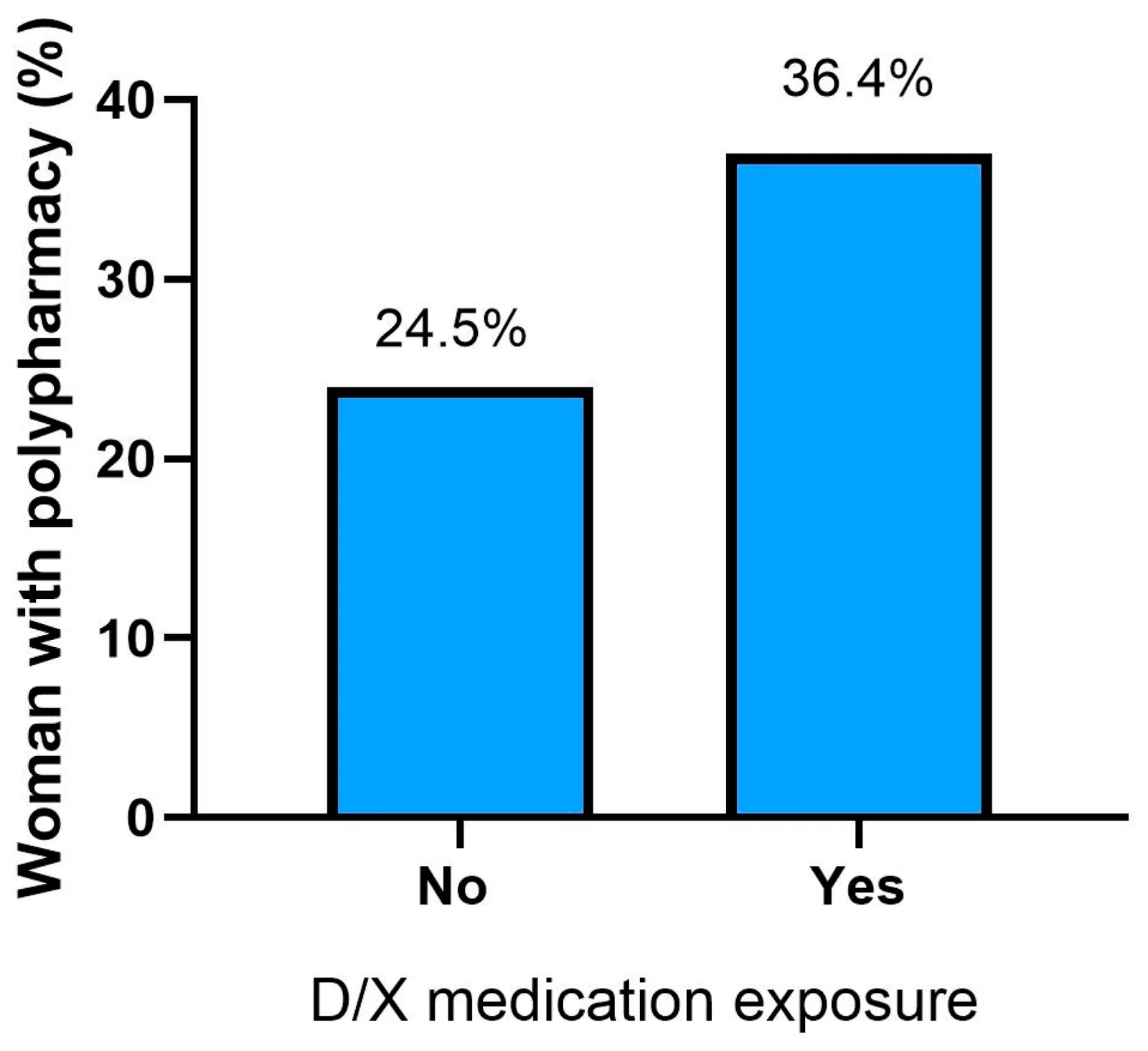
Proportion of women exposed to at least one former FDA pregnancy category D or X medication according to polypharmacy status. Polypharmacy was observed in 24.5% of women without D/X exposure and 36.4% of women with D/X exposure. Percentages were calculated within each D/X exposure group.

Exposure to at least one category D/X medication was significantly associated with polypharmacy. Among patients with polypharmacy (≥5 medications), 98 of 147 (66.7%) were exposed to at least one category D/X medication, compared with 171 of 322 (53.1%) patients without polypharmacy. Conversely, polypharmacy was observed in 98 of 269 (36.4%) women exposed to at least one category D/X medication, compared with 49 of 200 (24.5%) women without category D/X exposure (Figure 5). This difference was statistically significant (χ^2^ = 7.59, *p* = 0.006).

The distribution of medication exposures across the former FDA pregnancy risk categories was examined descriptively according to chronic disease status at the medication-exposure level, with each recorded medication exposure considered separately, after excluding uncategorized exposures. Among medication exposures recorded for women with chronic diseases, category D accounted for 15.1% of classified exposures, compared with 5.9% among those recorded for women without chronic diseases. Conversely, category X accounted for 12.7% of classified medication exposures among women without chronic diseases, compared with 8.1% among women with chronic diseases. The distribution of FDA pregnancy risk categories differed significantly between the two groups in the unadjusted Pearson chi-square analysis (χ^2^ = 47.0, df = 4, *p* < 0.001).

## 4. Discussion

This study characterized medication utilization patterns and exposure to potentially high-risk medications among women seeking counseling from a Teratology Information Service. The results indicated that a significant percentage of pregnant patients were exposed to high-risk medications (category D/X), with over fifty percent of the patients reporting at least one such exposure. Moreover, polypharmacy was markedly linked to increased exposure to high-risk medications.

The predominance of first-trimester consultations is not unexpected, as this period corresponds to organogenesis, when concerns regarding potential teratogenic effects are greatest [12]. The relatively low number of preconception consultations suggests that counseling before pregnancy remains underutilized, consistent with previous reports indicating limited uptake of preconception care despite its recognized importance in reviewing and optimizing medication use before conception [13,14]. The growing awareness of preconception counselling may contribute to reducing unnecessary drug exposure and anxiety during pregnancy.

Previous meta-analyses have shown that medication use during pregnancy is common, particularly when vitamin and mineral supplements are included [15]. In the present study, vitamin and mineral products were analyzed separately to avoid overestimating exposure to pharmacological agents with potential fetal risk. This approach allowed a more accurate evaluation of exposure to medications requiring teratogenic risk assessment.

Approximately two-thirds of the pregnant women seeking counseling had at least one documented chronic disease. As previously recommended, women with chronic diseases should ideally receive preconception counseling to allow optimization of pharmacotherapy before pregnancy rather than seeking risk assessment only after pregnancy has been recognized [16]. Because discontinuation of necessary medications may be associated with poorer maternal and fetal outcomes, individualized counseling is essential to ensure that treatment decisions are guided by evidence rather than by fear of teratogenicity.

Polypharmacy was significantly associated with exposure to at least one FDA category D/X drug in our cohort. Although a greater number of medications inevitably increases the likelihood of exposure to higher-risk agents, this finding emphasizes the importance of careful medication review in pregnant women receiving multiple therapies. Previous studies have demonstrated that polypharmacy during pregnancy is increasingly common, particularly among women with multimorbidity, and may increase the complexity of medication management and the potential for drug–drug interactions [4,6]. In the present study, polypharmacy (≥5 medications) was observed in 31.3% of pregnant women. Although direct comparisons are difficult because definitions of polypharmacy vary considerably across studies, this finding is within the range reported in previous systematic reviews [6]. Importantly, polypharmacy does not necessarily indicate inappropriate prescribing but rather reflects the complexity of managing maternal comorbidities during pregnancy. Nevertheless, each additional medication increases the need for systematic evaluation of potential fetal risks and possible drug–drug interactions. Our findings further suggest that pregnant women exposed to multiple medications represent a population requiring particular attention during teratogenic risk assessment.

More than half of category X exposures in our cohort were related to hormonal preparations. This finding suggests that a substantial proportion of category X exposure may have resulted from medications prescribed for gynecological or reproductive indications rather than from inadvertent exposure to classical teratogenic drugs. Consequently, the proportion of category X exposure should be interpreted in the clinical context and not solely as an indicator of inappropriate medication use during pregnancy. This observation also highlights the importance of interpreting pregnancy risk classifications together with the clinical indication and timing of exposure rather than considering the assigned risk category in isolation. Taken together, these findings reinforce the important role of Teratology Information Services in supporting individualized risk assessment during pregnancy. Rather than recommending universal discontinuation of medications, TIS counseling facilitates evidence-based decision-making by balancing maternal therapeutic needs against potential fetal risks.

Although the former FDA pregnancy risk categories remain useful for comparison with previous studies, they have important limitations because they oversimplify fetal risk and do not adequately consider gestational timing, dose, route of administration, or the consequences of untreated maternal disease. Accordingly, medication safety should be interpreted using comprehensive evidence rather than pregnancy risk categories alone.

The relatively small number of recorded exposures to folic acid-containing products also warrants consideration. Although folic acid supplementation is an important component of preconception and prenatal care, only 10 exposures to folic acid-containing products were identified in our consultation records. This finding should not be interpreted as evidence of low folic acid use among the women in our cohort. Because the study relied on retrospective records of consultations primarily focused on medication-related concerns, routinely used supplements may not have been consistently reported or documented, particularly when they were unrelated to the reason for consultation. Furthermore, some women may have sought teratology counseling following medication exposure before recognizing their pregnancy and may not yet have initiated folic acid supplementation at the time of consultation. Therefore, the relatively small number of recorded folic acid exposures may reflect both incomplete documentation and the timing of consultation in relation to pregnancy recognition, rather than indicating inadequate supplementation throughout pregnancy.

This study has several limitations. First, it was conducted at a single Teratology Information Service and included a selected population of women referred specifically for teratogenic risk assessment or medication counseling. This referral-based design introduces potential selection bias, as women with medication exposures that raise concerns about fetal safety or those requiring more complex pharmacotherapy may be more likely to receive specialized counseling. Consequently, the observed prevalence of category D/X exposure and polypharmacy may be higher than that in the general pregnant population and should not be interpreted as population-level estimates. From a public health perspective, these findings primarily characterize the medication-related concerns encountered in specialized teratology counseling rather than the overall burden of potentially high-risk medication exposure during pregnancy. Nevertheless, they may help identify areas in which medication review, risk communication, and access to evidence-based counseling could be strengthened. Population-based studies would be needed to determine the prevalence of these exposures among pregnant women more broadly. Second, medication exposure was determined from patient reports and available medical records, and medication adherence could not be verified. Third, fetal and neonatal outcomes were not systematically followed, precluding assessment of the relationship between medication exposure and pregnancy outcomes. Fourth, the retrospective design limited the availability of detailed information regarding the exact timing, dose, duration, and indication for some medication exposures. Fifth, the comparison of FDA pregnancy risk categories according to chronic disease status was conducted at the medication-exposure level. Because individual women could contribute multiple medication exposures, observations within the same woman may not have been independent. This potential within-woman correlation was not accounted for in the chi-square analysis and should be considered when interpreting the statistical significance of this association. Finally, the use of the former FDA pregnancy risk classification represents a methodological limitation, as these categories do not fully capture the complexity of medication safety during pregnancy. Nevertheless, they were used as a descriptive framework to enable standardized retrospective categorization and comparison with previous studies.

Despite these limitations, the study provides real-world data on medication exposure patterns among women referred for teratogenic risk assessment over a nine-year period. The simultaneous evaluation of medication utilization, polypharmacy, chronic disease, and exposure to potentially high-risk medications provides insight into the complexity of pharmacotherapy encountered in routine teratology counseling.

## 5. Conclusions

Medication exposure requiring individualized risk assessment was common among pregnant women referred to a Teratology Information Service, with more than half having been exposed to at least one medication classified as former FDA pregnancy category D or X. Polypharmacy was significantly associated with D/X exposure, while differences according to chronic disease status and the predominance of hormonal preparations among category X exposures highlighted the importance of interpreting pregnancy risk classifications within their clinical context. These findings support systematic medication review and individualized benefit–risk assessment, particularly for women receiving multiple medications, and reinforce the role of Teratology Information Services in evidence-based medication counseling before and during pregnancy.

## Figures and Tables

**Table 1:** Baseline characteristics of the study population.

Variable	Value
Total consultations	483
Pregnant women	469
Preconception counseling	14 (2.9%)
Maternal age (mean ± SD)	31.1 ± 5.5
Median age (range)	31 (16–46)
Gestational age (weeks)	9.69 ± 3.94
Pregnancy period	
First trimester	81.4%
Second trimester	18.6%
Third trimester	0
Mean number of medications	3.88 ± 2.72
Polypharmacy (≥5 drugs)	31.3%

## Data Availability

The data supporting the findings of this study are available from the corresponding author upon reasonable request.

## Abbreviations

ATC	Anatomical Therapeutic Chemical
FDA	Food and Drug Administration
LMP	Last Menstrual Period
PLLR	Pregnancy and Lactation Labeling Rule
SD	Standard Deviation
SmPC	Summary of Product Characteristics
TIS	Teratology Information Service
WHO	World Health Organization

## Appendix A

**Table A1:** Distribution of medications according to ATC classification.

ATC Classification	Counts	% of Total
A (Alimentary tract and metabolism)		13%
A01 STOMATOLOGICAL PREPARATIONS	8	0.4 %
A02 DRUGS FOR ACID RELATED DISORDERS	73	3.9 %
A03 DRUGS FOR FUNCTIONAL GASTROINTESTINAL DISORDERS	43	2.3 %
A04 ANTIEMETICS AND ANTINAUSEANTS	13	0.7 %
A06 DRUGS FOR CONSTIPATION	10	0.5 %
A07 ANTIDIARRHEALS, INTESTINAL ANTIINFLAMMATORY/ANTIINFECTIVE AGENTS	17	0.9 %
A08 ANTIOBESITY PREPARATIONS, EXCL. DIET PRODUCTS	1	0.1 %
A09 DIGESTIVES, INCL. ENZYMES	2	0.1 %
A10 DRUGS USED IN DIABETES	31	1.7 %
A11 VITAMINS	32	1.7 %
A12 MINERAL SUPPLEMENTS	10	0.5 %
A13 TONICS	1	0.1 %
A16 OTHER ALIMENTARY TRACT AND METABOLISM PRODUCTS	1	0.1 %
B (Blood and blood forming organs)		4.7%
B01 ANTITHROMBOTIC AGENTS	63	3.4 %
B03 ANTIANEMIC PREPARATIONS	24	1.3 %
C (Cardiovascular system)		4.7%
C01 CARDIAC THERAPY	2	0.1 %
C02 ANTIHYPERTENSIVES	10	0.5 %
C03 DIURETICS	1	0.1 %
C05 VASOPROTECTIVES	10	0.5 %
C07 BETA BLOCKING AGENTS	31	1.7 %
C08 CALCIUM CHANNEL BLOCKERS	6	0.3 %
C09 AGENTS ACTING ON THE RENIN-ANGIOTENSIN SYSTEM	20	1.1 %
C10 LIPID MODIFYING AGENTS	8	0.4 %
D (Dermatologicals)		1.6%
D01 ANTIFUNGALS FOR DERMATOLOGICAL USE	7	0.4 %
D02 EMOLLIENTS AND PROTECTIVES	2	0.1 %
D06 ANTIBIOTICS AND CHEMOTHERAPEUTICS FOR DERMATOLOGICAL USE	3	0.2 %
D07 CORTICOSTEROIDS, DERMATOLOGICAL PREPARATIONS	6	0.3 %
D10 ANTI-ACNE PREPARATIONS	12	0.6 %
G (Genito urinary system and sex hormones)		7.0%
G01 GYNECOLOGICAL ANTIINFECTIVES AND ANTISEPTICS	13	0.7 %
G02 OTHER GYNECOLOGICALS	5	0.3 %
G03 SEX HORMONES AND MODULATORS OF THE GENITAL SYSTEM	111	6.0 %
G04 UROLOGICALS	2	0.1 %
H (Systemic hormonal preparations, excl. sex hormones and insulins)		4.4%
H02 CORTICOSTEROIDS FOR SYSTEMIC USE	52	2.8 %
H03 THYROID THERAPY	29	1.6 %
J (Antiinfectives for systemic use)		11.6%
J01 ANTIBACTERIALS FOR SYSTEMIC USE	163	8.8 %
J02 ANTIMYCOTICS FOR SYSTEMIC USE	9	0.5 %
J04 ANTIMYCOBACTERIALS	8	0.4 %
J05 ANTIVIRALS FOR SYSTEMIC USE	29	1.6 %
J06 IMMUNE SERA AND IMMUNOGLOBULINS	1	0.1 %
J07 VACCINES	5	0.3 %
L (Antineoplastic and immunomodulating agents)		4.7%
L01 ANTINEOPLASTIC AGENTS	16	0.9 %
L03 IMMUNOSTIMULANTS	6	0.3 %
L04 IMMUNOSUPPRESSANTS	66	3.6 %
M (Musculoscelatal system)		14.7%
M01 ANTIINFLAMMATORY AND ANTIRHEUMATIC PRODUCTS	217	11.7 %
M02 TOPICAL PRODUCTS FOR JOINT AND MUSCULAR PAIN	3	0.2 %
M03 MUSCLE RELAXANTS	37	2.0 %
M04 ANTIGOUT PREPARATIONS	14	0.8 %
M05 DRUGS FOR TREATMENT OF BONE DISEASES	1	0.1 %
M09 OTHER DRUGS FOR DISORDERS OF THE MUSCULO-SKELETAL SYSTEM	1	0.1 %
N (Nervous system)		22.7%
N01 ANESTHETICS	15	0.8 %
N02 ANALGESICS	98	5.3 %
N03 ANTIEPILEPTICS	67	3.6 %
N04 ANTI-PARKINSON DRUGS	7	0.4 %
N05 PSYCHOLEPTICS	86	4.6 %
N06 PSYCHOANALEPTICS	138	7.4 %
N07 OTHER NERVOUS SYSTEM DRUGS	11	0.6 %
P (Antiparasitic products, insecticides and repellents)		1.9%
P01 ANTIPROTOZOALS	31	1.7 %
P02 ANTHELMINTICS	4	0.2 %
P03 ECTOPARASITICIDES, INCL. SCABICIDES, INSECTICIDES AND REPELLENTS	1	0.1 %
R (Respiratory system)		7.5%
R01 NASAL PREPARATIONS	15	0.8 %
R02 THROAT PREPARATIONS	4	0.2 %
R03 DRUGS FOR OBSTRUCTIVE AIRWAY DISEASES	46	2.5 %
R05 COUGH AND COLD PREPARATIONS	32	1.7 %
R06 ANTIHISTAMINES FOR SYSTEMIC USE	43	2.3 %
S (Sensory organs)		0.2%
S01 OPHTHALMOLOGICALS	3	0.2 %
V (Various)		1.3%
V03 ALL OTHER THERAPEUTIC PRODUCTS	1	0.1 %
V07 ALL OTHER NON-THERAPEUTIC PRODUCTS	1	0.1%
V08 CONTRAST MEDIA	22	1.2 %
